# Recovery from Resistance Exercise in Older Adults: A Systematic Scoping Review

**DOI:** 10.1186/s40798-023-00597-1

**Published:** 2023-07-03

**Authors:** Eleanor Jayne Hayes, Emma Stevenson, Avan Aihie Sayer, Antoneta Granic, Christopher Hurst

**Affiliations:** 1 AGE Research Group, Faculty of Medical Sciences, Translational and Clinical Research Institute, Newcastle University, Newcastle Upon Tyne, UK; 2 Faculty of Medical Sciences, Population Health Sciences Institute, Newcastle University, Cookson Building, 1St Floor, Newcastle Upon Tyne, UK; 3 NIHR Newcastle Biomedical Research Centre, Newcastle Upon Tyne Hospitals NHS Foundation Trust, Newcastle University, Newcastle Upon Tyne, UK

**Keywords:** Older adults, Resistance exercise, Exercise recovery, Scoping review, Exercise programming, Muscle soreness, Muscle strength, Delayed onset muscle soreness, Strength training, Muscle damage

## Abstract

**Background:**

Resistance exercise is recommended for maintaining muscle mass and strength in older adults. However, little is known about exercise-induced muscle damage and recovery from resistance exercise in older adults. This may have implications for exercise prescription. This scoping review aimed to identify and provide a broad overview of the available literature, examine how this research has been conducted, and identify current knowledge gaps relating to exercise-induced muscle damage and recovery from resistance exercise in older adults.

**Methods:**

Studies were included if they included older adults aged 65 years and over, and reported any markers of exercise-induced muscle damage after performing a bout of resistance exercise. The following electronic databases were searched using a combination of MeSH terms and free text: MEDLINE, Scopus, Embase, SPORTDiscus and Web of Science. Additionally, reference lists of identified articles were screened for eligible studies. Data were extracted from eligible studies using a standardised form. Studies were collated and are reported by emergent theme or outcomes.

**Results:**

A total of 10,976 possible articles were identified and 27 original research articles were included. Findings are reported by theme; sex differences in recovery from resistance exercise, symptoms of exercise-induced muscle damage, and biological markers of muscle damage.

**Conclusions:**

Despite the volume of available data, there is considerable variability in study protocols and inconsistency in findings reported. Across all measures of exercise-induced muscle damage, data in women are lacking when compared to males, and rectifying this discrepancy should be a focus of future studies. Current available data make it challenging to provide clear recommendations to those prescribing resistance exercise for older people.

## Key Points

- Understanding recovery from resistance exercise in older adults is essential for ensuring optimal exercise prescription, safety, and adherence to exercise programmes.
- A broad scope of markers of muscle damage have been reported, but study protocols and findings are inconsistent across the literature.
- Data focussing on recovery from resistance exercise in older females are sparse and should be a focus of future studies.

## Background

There is a growing body of literature recommending resistance exercise for the maintenance of muscle function in older adults including those living with sarcopenia and physical frailty [1–3]. Resistance exercise can increase muscular strength and power [4, 5], improve metabolic health [6], and reduce falls risk [7, 8] in older adults. These chronic adaptations are well documented, but the acute effects of resistance exercise, specifically changes associated with exercise-induced muscle damage (EIMD), in older people are less well characterised. These changes could be temporarily detrimental to an individual’s ability to carry out daily activities or may transiently increase falls risk [3, 9]. The high eccentric forces associated with resistance exercise combined with the unaccustomed load and volume at the beginning of a training programme [10, 11], mean it is well accepted that resistance exercise can induce EIMD. However, the duration and magnitude of its effects are highly variable, and depend both on the training variables [12–14] and individual characteristics [15–17], such as training intensity [12], training status [15], age [18], and sex [16]. Hence, a greater understanding of how function is affected in the days following a bout of resistance exercise is essential to inform better exercise prescription, and the formation of suitable recovery strategies for older adults.

The examination of a biopsy sample under a microscope is needed to directly quantify damage to the skeletal muscle. However, this is an invasive technique, which is not without risk. More importantly, the small amount of tissue obtained from a muscle biopsy may not accurately reflect the damage across the whole muscle. As such, indirect markers of muscle damage are often used to assess the extent to which individuals have been affected by intense or unaccustomed exercise. Currently, the magnitude of muscle strength loss is thought to be the best indicator of EIMD. However, a variety of other markers are also commonly used, including measures of muscle function and muscle power, muscle soreness, the cytokine response, the presence of circulating muscle proteins, and the immune cell response.

Declines in physical functioning after resistance exercise are a particularly important consideration in an older population [19]. This is because a decrease in muscle strength and function is one of the primary indicators of structural muscle damage but it is also directly relevant to the individual experiencing it. Older adults commonly have a lower baseline muscle function than their younger counterparts due to a number of age-related factors including the loss of muscle mass [20, 21], neuromuscular junction dysfunction [21, 22], changes in mitochondrial function [23, 24] and impairment of calcium storage and release [25]. Hence, any further decrease in physical function as a result of intense exercise may affect the safety and physical capability of the individual [26, 27]. This could have implications for older adults performing basic tasks of daily living. For example, it could become difficult to climb stairs, or it could increase their fall risk. Generally, the risk of falls following resistance exercise would not be a consideration when working with younger populations. However, in older adults this is a pertinent and often overlooked adverse outcome where the losses in muscle function and neuromuscular fatigue experienced after resistance exercise result in reduced muscle power across a variety of movement speeds and a decrease in an individual’s ability to maintain static and dynamic balance [9, 27], which may increase risk of falls.

Muscle soreness, or delayed onset muscle soreness (DOMS), is another commonly used marker of EIMD [11, 28]. Although the mechanisms underlying muscle soreness remain unclear, it has been proposed that the sensation results from several sources including damage to the muscle structure and connective tissue, disrupted calcium homeostasis, and the infiltration of inflammatory cells causing sensitisation of the nociceptors [11, 29]. Whilst muscle soreness does not correlate directly with the magnitude of damage within the muscle, it is an important factor to consider when characterising the exercise recovery process. Indeed, muscle soreness is likely the most prominent physical symptom of damage for the exercised individual, and may affect perceived recovery [30, 31] and willingness to perform further exercise [3]. Hence the extent of, and recovery time of, muscle soreness could have implications for adherence and exercise programming [3].

Other markers of EIMD can be measured in venous blood samples. These include circulatory markers of muscle damage and inflammatory cytokines [32–35]. Following EIMD, intracellular muscle proteins can leak in to the extracellular space due to membrane damage. Numerous muscle proteins can then be found in the plasma, but the most commonly used muscle damage biomarkers are creatine kinase (CK), myoglobin (Mb), and lactate dehydrogenase (LDH) [36].Whilst these are generally more variable, and therefore less reliable than other markers of muscle damage [37], they can be used alongside them to build a more complete picture of physiological disturbances. Alongside an increase in circulating muscle proteins there is also a strong inflammatory response to eccentric exercise that contributes to EIMD and is essential for regulating adaptation to resistance exercise. This response is highly orchestrated and involves both immune cells and associated cytokines (IL-1, IL-6, IL-10, TNF-α). The accumulation of inflammatory cells in the muscle tissue is considered an important indicator of EIMD [37]. However, the complexity of the inflammatory response makes it hard to draw parallels with histological damage.

A greater understanding of how these markers change following resistance exercise in older adults is needed. This will allow researchers and exercise practitioners to understand how EIMD and the related symptoms affect the daily lives of older adults. Recommendations can then be made to ensure optimal safety, efficacy, and continued engagement, whilst maximising the potential of resistance exercise. It is also possible that sex differences may exist in recovery from resistance exercise, although no review of this topic has been conducted [17]. Indeed, to the best of our knowledge, there has been no comprehensive review of recovery from resistance exercise in older adults to date. The broad range of outcomes that characterise exercise recovery, as well as the variation in the approaches to studying EIMD, make it challenging to pinpoint specific research questions that need resolving. Using a scoping review to identify the available evidence and examine how previous research in this area has been conducted will allow these gaps in the current knowledge to be determined. More specific questions relating to EIMD in older adults can then be addressed by systematic review and meta-analyses [38].

This scoping review therefore aimed to identify and provide an overview of the current literature surrounding EIMD, and recovery from resistance exercise in older adults. We aimed to establish; (a) which sub-groups of the older population have been studied; (b) how the exercise recovery process has been characterised; (c) what acute post-exercise effects of resistance training have been documented in older adults; (d) the time-course of exercise recovery in older adults and; (e) what variables (if any) have been shown to alter the exercise recovery process in this population.

## Methods

### General

This review protocol has previously been published [39] and is reported in accordance with the guidance from the Preferred Reporting Items for Systematic Reviews and Meta-Analyses extension for Scoping Reviews (PRISMA-ScR) [40]. The review reports on the current state of the literature regarding the acute effects of resistance exercise in older adults and follows the framework for scoping reviews first outlined by Arksey and O’Malley [41] and further refined by the Joanna Briggs Institute [42].

### Identifying Relevant Studies

The following electronic databases were searched using MeSH terms and free text: MEDLINE, Scopus, Embase, SPORTDiscus and Web of Science on 6th April 2021, and an updated search was performed on 23rd January 2023. In addition, reference lists of all identified articles were screened for additional studies.

The search strategy included terms related to the population of interest (i.e., adults, older adults, elderly) in combination with the exercise mode (i.e., resistance training, weight training, weight-lifting, resistance exercise) and the outcomes of interest (i.e., muscle damage, exercise recovery, muscle soreness, muscle function, muscle strength, isometric strength, creatine kinase, inflammation, perceived recovery). The full search strategy is included within the previously published protocol paper [39]. Within this review, older adults are defined to be those over 65 years of age. This is because within the United Kingdom the National Health Service (NHS) classifies older adults as those aged 65 years and over, with their physical activity guidelines reflecting this classification [43, 44].

### Study Selection

All eligible articles were uploaded to Zotero 5.0, where duplicate articles were removed. The authors conducted the initial screening of the articles. To ensure the suitability of the selected studies for the research objectives, two reviewers (EJH and CH) screened by title and abstract. Any excluded studies were reviewed by a third reviewer (AG). If the eligibility of a study was not clear from the abstract, a full-text article was obtained. Kappa coefficient between the two reviewers was 0.57 for title and abstract selection. Full-text screening of the subsequently selected articles was conducted by two reviewers independently (EJH and CH). Every effort was made to obtain full-text articles of the selected articles, including web searching, contacting the necessary authors, and consultation of a university librarian. Kappa coefficient was 0.70 for full-text selection. These values included cases where either reviewer marked an article to be further investigated and discussed. Discrepancies between reviewers was initially sought to be rectified by discussion. In the case of no resolution, a third reviewer (AG) was asked to determine a consensus.

### Charting of the Data

Data were extracted from all eligible studies using a standardised form to chart the data developed by three reviewers (EJH, CH, and AG). This form aimed to gather all the relevant information surrounding the exercise recovery process in older adults. Data were charted by one author (EJH) and checked by a second author (CH). Any disagreements were resolved first by discussion between the two reviewers or further adjudicated by the third (AG) if a unanimous decision was not made.

Information of interest included the following:

- *Study characteristics* year of publication, journal, aims and objectives of the study, study design, sample size, country of origin, study setting
- *Participant characteristics* population sampled, age (e.g., mean with standard deviation and range), sex (e.g., percentage of male/female participants), training status (e.g., untrained or resistance-trained).
- Resistance exercise intervention protocol (e.g., exercises performed, muscle groups used, training intensity, training volume, contraction type, and other relevant information).
- Outcome results (e.g., finding relevant to exercise recovery or exercise-induced muscle damage)
- The time frame of outcome measures (e.g., at what time-points were data collected in relation to the resistance exercise protocol)
- Presence of any comparison groups (e.g., young adults or a nutritional intervention)
- Key relevant findings and conclusions

## Results

The results of this review are organised by themes that were recurrent throughout the literature, or that the authors deemed relevant for advancing current knowledge. Age differences in recovery from resistance exercise are presented first, followed by symptoms of EIMD, biological markers of muscle damage, and finally, factors that may affect EIMD and exercise recovery in older adults. For the purpose of this review, circulating muscle proteins includes creatine kinase (CK), myoglobin, and lactate dehydrogenase (LDH) as these are the most commonly reported within the literature. Where multiple articles were found that reported data from the same study or data set, these have been included separately to provide a comprehensive overview of the published literature.

### Study Selection

**Fig. 1:**
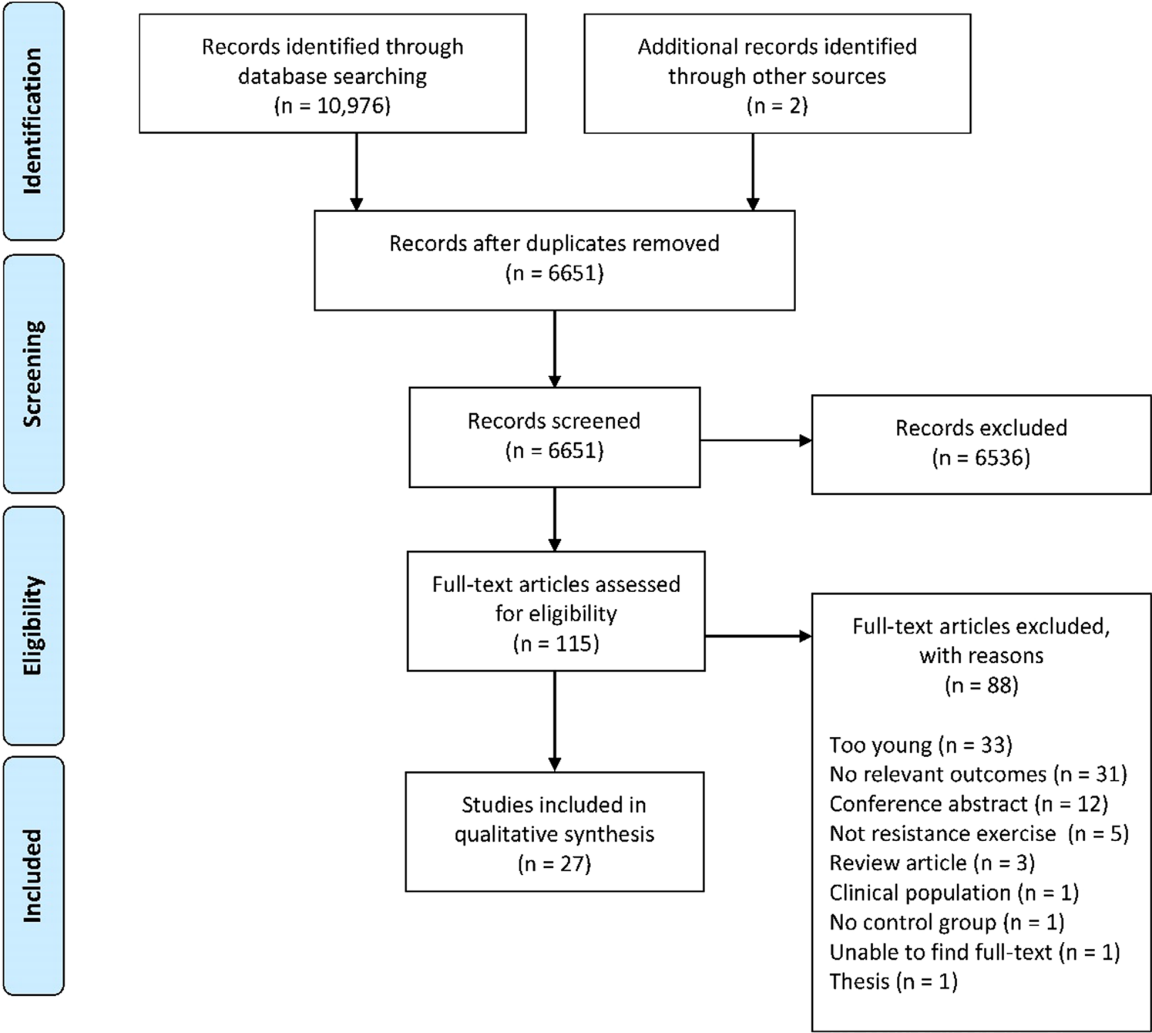
PRISMA flow diagram

Following the initial database search, 10,976 records were identified (Fig. 1). Once duplicates were removed, 6651 titles and abstracts remained, and were screened for inclusion, resulting in 115 full-text articles being screened. Of these, 88 were excluded and 27 were included for qualitative synthesis.

### Age Differences in Recovery from Resistance Exercise

#### The Effect of Age on Exercise-Induced Muscle Damage in Men

Six studies were found that reported the effects of age on exercise-induced muscle damage (EIMD) in older men exclusively (Table 1). Four of the studies reported on symptoms of muscle damage such as physical functioning and muscle soreness [45–47] and one reported on the inflammatory response [48]. Three studies originated from the same research group, and used the same eccentric exercise protocol of the elbow flexors (6 × 5 repetitions at 40% maximal isometric strength) [45–47]. The remaining two employed a concentric exercise protocol for the lower limbs [48, 49].

**Table 1:** The effect of age on exercise-induced muscle damage in men

Study	Subjects	Age (years)	Exercise	Main outcomes	Effect of age on magnitude of change	Effect of age on time to recovery	Other findings
Lavender and Nosaka [45]	20 healthy males (10 young, 10 older)	19 ± 0, 71 ± 2	6 × 5 reps eccentric contractions of elbow flexors 40% maximal isometric strength	MIVC Muscle soreness CK Mb Range of motion Limb circumference	↓* ↓* ↓* ↓* ↓* ↔	Not recovered (240-h) ↓ Not given Not given ↓ Not recovered (240-h)	
Lavender and Nosaka [46]	18 healthy males (10 young, 8 older)	20 ± 2, 71 ± 4	6 × 5 reps eccentric contractions of elbow flexors 40% maximal isometric strength	MIVC Muscle soreness CK Mb Range of motion Limb circumference	↓* ↓* ↓* ↓* ↓* ↔	Not recovered (96-h) ↓ Not recovered (96-h) Not recovered (96-h) Not recovered (96-h) Not recovered (96-h)	Repeated bout (4-wks) did not confer a protective effect for MIVC but it did for soreness and Mb
Przybyla et al. [48]	34 healthy males (17 young, 17 older)	32 ± 7, 71 ± 5	3 × 8 reps for bilateral leg press, leg curl, and leg extension 80% 1-RM. Plus 4th set to failure	Macrophage number Cytokine expression	↓ ↓	N/A N/A	At rest, older adults had higher levels of IL-1β, IL-1RA, and IL-10
Lavender and Nosaka [47]	32 healthy males (10 young, 12 middle-aged, 10 older)	20 ± 2, 48 ± 7, 71 ± 4	6 × 5 reps eccentric contractions of elbow flexors 40% maximal isometric strength	MIVC	↓*	Not recovered (120-h)	No changes in force fluctuations at any intensity (30%, 50%, 80% MIVC)
Nikolaidis et al. [49]	20 healthy males (10 young, 10 older)	22 ± 4, 67 ± 5	5 × 15 reps of back squat 75% 1-RM on a Smith machine	MIVC Muscle soreness CK Range of motion Joint position sense	↓* ↔ ↔ ↔ ↔	N/A N/A N/A N/A N/A	

*1-RM* one-repetition maximum, *MIVC* maximal isometric voluntary contraction, *CK* creatine kinase, *Mb* myoglobin, *h *hours, *wks* weeks, ↓ decrease, ↔ no change

*Significantly different from baseline, reps repetitions

Four out of the five studies reported at least one variable that had a significantly smaller magnitude of change in older adults compared to the young group [45–47, 49]. Przybyla et al. [48] did not record any significant differences between groups, but the older group (71 ± 5) did tend to have a smaller increase in macrophage number and cytokine response post-exercise compared to the younger group (32 ± 7). None of the studies observed full recovery of symptoms of EIMD, despite some studies continuing for 10-days post exercise.

#### The Effect of Age on Exercise-Induced Muscle Damage in Women

Three articles aimed to understand the effect of age on EIMD in women exclusively (Table 2). Clarkson and Dedrick[50], and Dedrick and Clarkson [51] reported one study in two separate papers, and hence, only two unique studies are reported.

**Table 2:** The effect of age on exercise-induced muscle damage in women

Study	Subjects	Age (years)	Exercise	Main outcomes	Effect of age on magnitude of change	Effect of age on time to recovery	Other findings
Clarkson and Dedrick [50]	20 healthy females (10 young, 10 older)	24 ± 3, 67 ± 5	24 reps eccentric contractions of elbow flexors 115% maximal isometric strength	Relaxed elbow angle Flexed elbow angle Muscle soreness CK	↑* ↔ ↔ ↔	↑ ↔ ↔ Not recovered (120-h)	
Dedrick and Clarkson [51]	20 healthy females (10 young, 10 older)	24 ± 3, 67 ± 5	24 reps eccentric contractions of elbow flexors 115% maximal isometric strength	MIVC Muscle soreness Reaction time Movement time	↑ ↔ ↔ ↔	↑ ↔ ↔ ↔	No significant difference in magnitude of strength loss, but older recovered more slowly
Ploutz-Snyder et al. [52]	12 healthy females (6 young, 6 older)	23 ± 4, 66 ± 5	10 × 10 reps unilateral eccentric contractions of knee extensors 75% eccentric 1-RM	Concentric 1-RM Eccentric 1-RM	↑* ↑*	↑ ↑	12-wk RT programme attenuated declines in muscular strength, losing only 14% (Con) and 12% (Ecc) of 1-RM strength

*reps* repetitions, *1-RM* one-repetition maximum, *MIVC* maximal isometric voluntary contraction, *CK* creatine kinase, *Con* concentric, *Ecc* Eccentric, *h* hours, *wk* week, ↑ increase, ↔ no change

*Significantly different from baseline

Clarkson and Dedrick [50], and Dedrick and Clarkson [51] both used an eccentric resistance exercise protocol consisting of 24 repetitions of the elbow flexors at 115% maximal isometric strength in twenty healthy women. Ploutz-Snyder et al. [52] also used eccentric contractions, but their exercise protocol was ten sets of ten repetitions of the knee extensors at 75% eccentric 1-RM.

The older women (67 ± 5) within the Clarkson and Dedrick [50], and Dedrick and Clarkson [51] study experienced greater decreases in maximal isometric strength [51] and, and also recovered these measures more slowly than the younger group (24 ± 3) in the days following exercise. No differences in muscle soreness or creatine kinase were observed between the two groups. Similar results were observed by Ploutz-Snyder et al. [52], with concentric and eccentric 1-RM both decreasing more in older adults, and taking longer to recover after eccentric exercise of the knee extensors.

### Symptoms of Exercise-Induced Muscle Damage

#### Physical Function

Fourteen studies measured physical function in adults over 65 years of age (Table 3). The most common measure of physical functioning was maximal voluntary contraction (MVC) (12/14 studies). One study used one repetition maximum (1-RM) to assess strength [52], and one study used neither MVC nor 1-RM [53], instead measuring counter-movement jump height, hand grip strength, and seated medicine ball throw performance. The Timed-Up-and-Go test (TUG), often used to assess both mobility, and static and dynamic balance, was used in two of the articles [12, 54] alongside other measures of performance.

**Table 3:** Physical Function

Study	Subjects	Age (years)	Exercise	Time points	Marker	Results			Other findings
						Time to peak	Magnitude of change	Time to recovery	
Dedrick and Clarkson [51]	20 healthy females (10 young, 10 older)	24 ± 3, 67 ± 5	24 reps eccentric contractions of elbow flexors 115% maximal isometric strength	Baseline, 24-, 48-, 72-, 96-, 120-h	MIVC elbow flexor	24—48-h	↓12 Nm (42%)*#	Not recovered	
Ploutz-Snyder et al. [52]	12 healthy females (6 young, 6 older)	23 ± 4, 66 ± 5	10 × 10 reps unilateral eccentric contractions of knee extensors 75% eccentric 1-RM	Baseline, 24-, 72-, 96-, 168-, 216-, 264-h	1-RM CON 1-RM ECC	24-h 24-h	↓24%* ↓27%*	168-h 72-h	Strength deficit after RT reduced after 12-wks training
Ferri et al. [55]	9 healthy males	72 ± 4	10 × 10 reps seated calf raises 70% 1-RM	Baseline, 1-, 48-, 96-, 144-h	MIVC ankle flexor Maximal voluntary torque at; -60° s-^1^ 60° s-^1^ 120° s-^1^	N/A 1-h N/A 1-h	↔ ↓ 8.4 Nm (9%)* ↔ ↓ 4.5 Nm (16%)*	N/A 24-h N/A 24-h	Neuromuscular fatigue not exercise-induced muscle damage
Lavender & Nosaka [45]	20 healthy males (10 young, 10 older)	19 ± 0, 71 ± 2	6 × 5 reps eccentric contractions of elbow flexors 40% maximal isometric strength	Baseline, 0-, 1-, 24-, 48-, 72-, 96-, 120-, 168-, 240-h	MIVC elbow flexor	0-h	↓48%*#	Not recovered	MIVC was 87% of baseline values at 10-d
Lavender and Nosaka [46]	18 healthy males (10 young, 8 older)	20 ± 2, 71 ± 4	6 × 5 reps eccentric contractions of elbow flexors 40% maximal isometric strength	Baseline, 0-, 24-, 48-, 72-, 96-h	MIVC elbow flexor	0-h	↓48%*#	Not recovered	Repeated bout did not significantly attenuate decreases in MIVC
Lavender and Nosaka [47]	32 healthy males (10 young, 12 middle-aged, 10 older)	20 ± 2, 48 ± 7, 71 ± 4	6 × 5 reps eccentric contractions of elbow flexors 40% maximal isometric strength	Baseline, 0-, 24-, 48-, 72-, 96-, 120-h	MIVC elbow flexor	0-h	↓49%*#	Not recovered	Ageing does not affect force fluctuations before or after eccentric exercise
Chen et al. [56]	26 healthy males	66 ± 5	6 × 10 reps maximal eccentric contractions of knee extensors	Baseline, 0-, 24-, 48-, 72-, 96-, 120-h	MVC-CON 30° s-^1^ knee extensors	0-h	↓28%*#	Not recovered	Low intensity eccentric bout 7-d prior reduced deficits in MVC
Buford et al. [57]	30 healthy adults (15 young, 15 older). Each group 5 females, 5 males	23 ± 4, 76 ± 5	150 reps unilateral eccentric contractions of plantar flexors 110% 1-RM	Baseline, 48-, 168-h	Maximal voluntary torque plantar flexors	48-h	↓20 Nm (14%)#	Not recovered	Old and young similar
Nikolaidis et al. [49]	20 healthy males (10 young, 10 older)	22 ± 4, 67 ± 5	5 × 15 reps of back squat 75% 1-RM on a Smith machine	Baseline, 48-h	MIVC knee extensors	N/A	↓31 Nm (-23%)*#	N/A	
Orssatto et al. [12]	22 healthy adults. 7 females, 15 males. Two groups	66 ± 5, 67 ± 5	3 × failure 70% or 95% 5-RM of leg press and leg curl	Baseline, 0-, 24-, 48-, 72-h	Maximal voluntary peak torque knee extensors Timed up and go CMJ Stair Ascent Stair Descent	0-h 0-h 0-h 72-h N/A	↓14%*# (G70) ↓17%*# (G95) ↑ 2%*# (G70) ↑ 6%*# (G95) ↓8%*# (G70) ↓11%*# (G90) ↑6%*# (G70) ↑4%*# (G95) ↔	24-h Not recovered 24-h 24-h 24-h 24-h Not recovered Not recovered N/A	Groups are 70% 5-RM (G70), and 95% 5-RM (G95)
Sorensen et al. [59]	19 healthy adults (11 young, 8 older). 4 females in young group	22 ± 2, 71 ± 7	300 reps maximal eccentric contractions of the knee extensors	Baseline, 24-, 72-h	MVC-CON 60° s-1 knee extensors Peak isokinetic power	0-h 0-h	↓34%*# ↓35%*#	72-h 24-h	Unable to recruit older women able to perform exercise
Marques et al. [53]	31 institutionalized adults. 14 males, 17 females	79 ± 7	2 or 4 sets of 5 CMJ. 3 sets of 6 or 12 reps 2 kg SMBT (seated medicine ball throw) 3 sets of 8 or 15 reps of leg-press and chest-press 65% 1-RM 3 sets of 6 or 12 reps 5 kg chair-squat	Baseline, 5-min	SMBT CMJ Hand-grip strength	N/A N/A N/A	↔low volume ↓3%* high volume ↓5% low volume ↓ 8%* high volume ↑3%* low volume ↓1% high volume	N/A N/A N/A	
Skarabot et al. [58]	33 healthy adults (12 young, 11 older). 2 and 3 females respectively	27 ± 5, 66 ± 4	10 sets of 6 reps maximal eccentric contractions of dorsi-flexions	Baseline, 0-, 24-, 72-h	MIVC knee extensors	0-h	↓ 22%*	72-h	Repeated bout protective
Naderi et al. [54]	78 healthy adults	66 ± 3	4 sets of 10 reps of 3 exercises (standing calf raise with DB, standing and seated calf raise with machine) 75% 1-RM	Baseline, 24-, 48-, 72-h	MVC-CON 60° s-1 plantar flexors Timed up and go	48-h 48-h	↓12 Nm (36%)*# ↑2 s (18%)*#	Not recovered Not recovered	Massage attenuated declines in strength and TUG
Rodriguez-Lopez et al. [67]	15 healthy adults (9 males, 6 females)	73.6 ± 3.8	6 × 6 reps leg press 80% of 1-RM (heavy) 6 × 12 reps leg press 40% of 1-RM (light)	Baseline, 0-h	MIVC knee extensors 5 Chair Stands	N/A	↓ 24.8 Nm (6%)*# ↑ 0.4 s (5%)*#	N/A	No effect of load

*1-RM* one-repetition maximum, *5-RM* five repetition maximum, *DB* dumbbell, *CMJ* countermovement jump, *SMBT* seated medicine ball throw, *MIVC* maximal isometric voluntary contraction, *CK* creatine kinase, *Mb* myoglobin, *CON* concentric, *ECC* eccentric, *h* hours, *d* days, *wks* weeks, *RT* resistance training, ↑ increase, ↓ decrease, ↔ no change

#Data extracted from figures

*Significantly different from baseline,

Studies that assessed function of the elbow flexors tended to report larger decreases in strength than those that assessed function of the lower limbs. For example, decreases in elbow flexor MVC after exercise ranged from 42 to 49% [47, 51], whilst decreases in plantar flexor and knee extensor strength ranged from 9 to 36% [54, 55]. Of the four studies that measured elbow flexor strength [45–47, 51], three were conducted by the same research group using the same exercise protocol [45–47]. Time to complete the TUG test varied between the two studies, with one reporting a 2% increase in time [12], and the other 18% [54]. Declines in muscle function tended to peak from 0 to 48-h, and in most studies took over 72-h to fully recover. In some instances, the total length of the study was not long enough to record total recovery of physical functioning [12, 45–47, 51, 54, 56, 57].

Of the four articles identified that involved a repeated bout within their protocol, three of the four suggested a protective effect of repeated exercise [52, 56, 58], whereas one study found it did not significantly lessen decreases in MVC [46]. Certain recovery strategies reduced muscle damage within older adults. Naderi et al. [54] found that massage was effective for attenuating declines in MVC and the TUG test at 24-, 48-, and 72-h following concentric exercise of the plantar flexors. Within the same study, cold water immersion was also found to attenuate deteriorations in the time to complete the TUG test at 48-h [54].

#### Muscle Soreness

Nine studies were identified that measured muscle soreness (pain) following resistance exercise (Table 4). Of these nine studies, seven involved eccentric exercise [45, 46, 50, 51, 56, 57, 59], seven included a younger group for comparison [45, 46, 49–51, 57, 59], and five included females in the sample [50, 51, 54, 57, 59]. All of the included studies used a visual analogue scale to assess perceived muscle soreness in the exercised limb. Due to slight discrepancies in the scales used to assess perceived soreness, it is difficult to provide an absolute value for the magnitude of change, but the identified studies all appeared to report only mild increases in soreness. Several studies reported that perceived muscle soreness was significantly lower in older adults when compared to a younger group [45, 46, 57], whilst others reported no effect of age [50, 51, 59]. Muscle soreness in older adults appears to peak at 24–48 h following damaging exercise, and is largely recovered ~ 3–5 days following exercise. Two studies provided evidence for reduced soreness after repeated exercise bouts [50, 56], and one study found that both massage and cold-water immersion could reduce soreness ratings 48 h after exercise [54]

**Table 4:** Muscle Soreness

Study	Subjects	Age (years)	Exercise	Time points	Scale	Results			Other findings
						Time to peak	Magnitude of change	Time to recover	
Clarkson and Dedrick [50]	20 healthy females (10 young, 10 older)	24 ± 3, 67 ± 5	24 reps eccentric contractions of elbow flexors 115% maximal isometric strength	Baseline, 24-, 48-, 72-, 96-, 120-h	1 (no pain) to 10 (very painful)	48-h	↑3 points*#	72-h	Older peaked 24-h after young Repeated bout (7-d) reduced pain rating
Dedrick and Clarkson [51]	20 healthy females (10 young, 10 older)	24 ± 3, 67 ± 5	24 reps eccentric contractions of elbow flexors 115% maximal isometric strength	Baseline, 24-, 48-, 72-, 96-, 120-h	1 (no pain) to 10 (very painful)	48-h	↑2 points*#	96-h	Older peaked 24-h after young
Lavender and Nosaka [46]	20 healthy males (10 young, 10 older)	19 ± 0, 71 ± 2	6 × 5 reps eccentric contractions of elbow flexors 40% maximal isometric strength	Baseline, 0-, 24-, 48-, 72-, 96-, 120-, 144-, 168-h	VAS: 0 (no pain) to 50 (extreme pain) mm	24–48-h	↑19 mm*#	120-h	Older had smaller increase compared to young
Lavender and Nosaka [46]	18 healthy males (10 young, 8 older)	20 ± 2, 71 ± 4	6 × 5 reps eccentric contractions of elbow flexors 40% maximal isometric strength	Baseline, 0-, 24-, 48-, 72-, 96-h	VAS: 0 (no pain) to 50 (extreme pain) mm	24–48-h	↑15 mm*#	96-h	Older had smaller increase compared to young Repeated bout effect more prominent in young
Chen et al. [56]	26 healthy males	66 ± 5	6 × 10 reps maximal eccentric contractions of knee extensors	Baseline, 0-, 24-, 48-, 72-, 96-, 120-h	VAS: 0 (not sore at all) to 100 (very, very sore) mm	48-h	↑12 mm*#	72-h	Performance of sub-maximal exercise 7-d prior reduced soreness by 6-mm at 48-h
Buford et al. [57]	30 healthy adults (15 young, 15 older). Each group 5 females, 5 males	23 ± 4, 76 ± 5	150 reps unilateral eccentric contractions of plantar flexors 110% 1-RM	Baseline, 48-, 168-h	VAS: 0 (no soreness) to 100 (extreme soreness) mm	48-h	↑28 mm*#	168-h	Older had smaller increase compared to young
Nikolaidis [49]	20 healthy males (10 young, 10 older)	22 ± 4, 67 ± 5	5 × 15 reps of back squat 75% 1-RM on a Smith machine	Baseline, 48-h	1 (normal) to 10 (very sore)	N/A	↑ 6 points*#	N/A	
Sorensen et al. [59]	19 healthy adults (11 young, 8 older). 4 females in young group	22 ± 2, 71 ± 7	300 reps maximal eccentric contractions of the knee extensors	Baseline, 24-, 72-h	VAS: 0 (no pain) to 100 (unbearable pain) mm	24-h	↑ 30 mm*#	Not recovered	No significant difference between young and old
Naderi et al. [54]	78 healthy adults	66 ± 3	4 sets of 10 reps of 3 exercises (standing calf raise with DB, standing and seated calf raise with machine) 75% 1-RM	Baseline, 24-, 48-, 72-h	VAS: 0 (no pain) to 100 (extreme pain) mm	48-h	↑ 49 mm (passive recovery)*#	Not recovered	Both massage and cold water immersion after exercise reduced soreness by 10-mm at 48-h

*1-RM* one-repetition maximum, *reps* repetitions, *DB* dumbbell, *VAS* visual analogue scale, *h* hours, *d* days, *DB* dumbbell, ↑ increase

^#^Data extracted from figures

*Significantly different from baseline,

#### Risk of Falls

Only two studies assessed balance and fall risk after an acute bout of resistance exercise in older adults [54, 60] (Table 5). Both studies included male and female participants and performed concentric resistance exercise of the lower limbs. Moore et al. [60] measured postural stability before and immediately after resistance exercise. The most recent study from Naderi et al. [54] provided data on three variables that are useful for assessing falls risk every 24-h for 72-h after resistance exercise of the plantar flexors. These variables include centre of pressure (COP) sway, joint ankle position error, and fear of falling [54]. The peak change in all three variables was statistically significant compared to baseline, and was observed at 48-h after exercise. This study also demonstrated that massage could attenuate increases in indicators of fall risk following muscle damaging exercise. Specifically, massage significantly reduced COP sway at 48–72 h, joint position error at 24-, 48-, and 72-h, and fear of falling at 24- and 72-h compared to passive recovery. In contrast, cold water immersion was found only to improve fear of falling at 72-h when compared to passive recovery.

**Table 5:** Risk of Falls

Study	Subjects	Age (years)	Exercise	Time-points	Outcome measure	Effect on falls risk	Other findings
Moore et al. [60]	21 healthy adults. 5 males, 16 females	71 ± 4	10–12 reps each of knee extension, ankle dorsiflexion, ankle plantar flexion, hip abduction, knee flexion	Baseline, 0-h	Diffusion co-efficient analysis Critical point analysis	↔ ↑*	
Naderi et al. [54]	78 healthy adults	66 ± 3	4 sets of 10 reps of 3 exercises (standing calf raise with DB, standing and seated calf raise with machine) 75% 1-RM	Baseline, 24-, 48-, 72-h	COP Sway Ankle joint position error Fear of falling	↑ 45% (48-h)* ↑ 81% (48-h)* ↑ 27% (48-h)*	Massage reduced COP sway at 48–72-h Massage reduced joint position error at 24-, 48-, and 72-h Massage improved fear of falling at 24- and 72-h. Cold water immersion improved fear of falling at 72-h

*1-RM* one-repetition maximum, reps repetitions, *COP* centre of pressure, *DB* dumbbells, h hours, ↑ increase, ↔ no change

*Significantly different from baseline

### Biological Markers of Muscle Damage

#### Circulating Muscle Proteins

Fifteen studies reported the acute effect of resistance exercise on circulating muscle proteins (Table 6). Four of these studies were conducted exclusively in females [50, 61–63], seven were conducted in males [45, 46, 49, 55, 56, 64, 65], and four were conducted with a mixed sample [57, 58, 66, 67]. Seven of the studies included a younger group of adults for comparison [45, 46, 49, 50, 57, 58, 66]. Eleven of the studies involved an eccentric exercise protocol [45, 46, 50, 56–58, 61–63, 65, 66], three of which were solely using elbow flexors [45, 46, 50], one used an eccentric-concentric protocol of the knee extensors [66]. The remainder used concentric protocols involving the whole body or lower limbs [49, 55, 64, 67].

**Table 6:** Circulating Muscle Proteins

Study	Subjects	Age (years)	Exercise	Time points	Marker	Results			Other findings
						Time to peak	Magnitude of change	Time to recovery	
Clarkson and Dedrick [50]	20 healthy females (10 young, 10 older)	24 ± 3, 67 ± 5	24 reps eccentric contractions of elbow flexors 115% maximal isometric strength	Baseline, 24-, 48-, 72-, 96-, 120-h	CK	120-h	↑180 IU/L (273%)*#	Not recovered	
Ferri et al. [55]	9 healthy males	72 ± 4	10 × 10 reps seated calf raises 70% 1-RM	Baseline, 1-, 48-, 96-, 144-h	CK Mb LDH	48-h 1-h 48-h	↑52 U/L (60.3%)* ↑19 U/L (73.2%)* ↑17 U/L (5.3%)*	96-h 48-h 96-h	
Lavender and Nosaka [45]	20 healthy males (10 young, 10 older)	19 ± 0, 71 ± 2	6 × 5 reps eccentric contractions of elbow flexors 40% maximal isometric strength	Baseline, 0-, 24-, 48-, 72-, 96-, 120-, 144-, 168-h	CK Mb	96—120-h 96—120-h	↑ 1996 IU/L (1358%)*# Baseline not given*	Not given Not given	Peak CK and Mb higher in young
Lavender and Nosaka [46]	18 healthy males (10 young, 8 older)	20 ± 2, 71 ± 4	6 × 5 reps eccentric contractions of elbow flexors 40% maximal isometric strength	Baseline, 0-, 24-, 48-, 72-, 96-h	CK Mb	96-h 96-h	↑1657 IU/L (1641%)*# ↑ 406 ng/ml (824%)*#	Not recovered Not recovered	Increases in CK and Mb higher in young Repeated bout significantly attenuated increases in Mb
Thalacker-Mercer et al. [66]	39 healthy adults (19 younger, 20 older)	37 ± 1, 73 ± 1	9 × 10 reps bilateral, concentric-eccentric knee extension 40% MIVC	Baseline, 24-h	CK	N/A	↑54 U/L (46%)*	Not recovered	
Chen et al. [56]	26 healthy males	66 ± 5	6 × 10 reps maximal eccentric contractions of knee extensors	Baseline, 0-, 24-, 48-, 72-, 96-, 120-h	CK Mb	96-h 96-h	↑ 1473 IU/L (1009%)*# ↑254 ug/L (726%)*#	Not recovered	Previous sub-max exercise significantly attenuated increases in CK and Mb
Funghetto et al. [61]	90 obese females	69 ± 6	7 × 10 reps eccentric bilateral knee extension isoinertial machine with a load corresponding to 110% of 10-RM	Baseline, 0-, 3-, 24-, 48-h	CK	24—48-h	↑39- 47 IU/L (42–44%)*	Not recovered	Only GG allele group had a peak significantly higher than baseline
Buford et al. [57]	30 healthy adults (15 young, 15 older). Each group 5 females, 5 males	23 ± 4, 76 ± 5	150 reps unilateral eccentric contractions of plantar flexors 110% 1-RM	Baseline, 48-, 168-h	CK	48-h	↑0.14 (log) IU/L	Not recovered	Older peaked later than young
Tajra et al. [62]	90 obese females	69 ± 6	7 × 10 reps eccentric bilateral knee extension isoinertial machine with a load corresponding to 110% of 10-RM	Baseline, 0-, 3-, 24-, 48-h	CK	48-h	↔(non-responders) ↑ 202 U/L (157%)*# (high responders)	N/A Not recovered	Evidence for high and low responders to CK
Nikolaidis [49]	20 healthy males (10 young, 10 older)	22 ± 4, 67 ± 5	5 × 15 reps of back squat 75% 1-RM on a Smith machine	Baseline, 48-h	CK	N/A	↑ 1534 IU/L (1112%)*#	N/A	No difference between young and older
Cornish et al. [64]	11 healthy males	72 ± 5	144 reps at 60% 1-RM OR 120 reps at 72% 1-RM OR 108 reps at 80% 1-RM of chest press, shoulder press, seated row, leg press, leg extension, and plantar flexion	Baseline, 0-, 3-, 6-, 24- 48-h	Mb	3—6-h	↑20 ng/ml (96%)*#	48-h	No effect of intensity on Mb
Pereira et al. [65]	28 healthy males	GG genotype: 71 ± 4 CC/CG genotype: 72 ± 4	10 × 7 reps of eccentric contractions of knee flexors and extensors	Baseline, 0-, 3-, 24-, 48-h	CK	24-h	↑85 U/L (57%)*#	Not recovered	No difference between genotypes
Skarabot et al. [58]	33 healthy adults (12 young, 11 older). 2 and 3 females respectively	27 ± 5, 66 ± 4	10 × 6 reps maximal eccentric contractions of dorsi-flexions	Baseline, 0-, 24-, 72-h	CK	24-h	↑108 IU/L (111%)*	Not recovered	Repeated bout significantly attenuated increases in CK
Rodriguez-Lopez et al. [67]	15 healthy adults (9 males, 6 females)	73.6 ± 3.8	6 × 6 reps leg press 80% of 1-RM (heavy) 6 × 12 reps leg press 40% of 1-RM (light)	Baseline, 0-, 24-h	CK LDH	24-h N/A	↑59 U/L (1083%)* ↔	N/A	No effect of load
De Sousa Neto et al. [63]	88 obese females	69 ± 6	7 × 10 reps eccentric bilateral knee extension isoinertial machine with a load corresponding to 110% of 10-RM	Baseline, 0-, 3-, 24-, 48-h	CK	48-h		Not recovered	No effect of muscle quality

*1-RM* one-repetition maximum, *reps* repetitions, *CK* creatine kinase, *Mb* myoglobin, *LDH* lactate dehydrogenase, *h* hours, ↑ increase, ↔ no change

^#^Data extracted from figures

*Significantly different from baseline

Creatine kinase was the mostly widely reported circulating muscle protein, being reported as a marker of muscle damage in 14/15 studies. Time to peak concentration of CK ranged from 24 to 120 h [45, 50, 58, 65], and was not recovered in the studies, with the exception of Ferri et al. [55], where CK returned to baseline at 96-h. The magnitude of increase of CK after exercise ranged from 46% [66] to 1641% [46] increase from baseline values.

Myoglobin (Mb) levels after resistance exercise in older adults were reported in five studies [45, 46, 55, 56, 64], with the studies reporting peaks from 1-h [55], to 120-h [45]. The increase of Mb ranged from 73% [55], to 824% [46]. One study did not provide a baseline value for Mb, and so an absolute or relative increase could not be calculated [45]. Lactate dehydrogenase (LDH) was reported by two studies [55, 67] where it reached a peak increase of 17 IU/L (5%) at 48-h, and recovered to baseline by 96-h.

#### Cytokines

Twelve studies investigated the effect of resistance exercise on the acute cytokine response in older adults (Table 7). The most common cytokine to be reported in the literature was interleukin-6 (IL-6) (10/12 studies), followed by tumor-necrosis-factor-α (TNF-α)(3/12), interleukin-1β (IL-1β)(3/12), interleukin-10 (IL-10) (2/12), and interleukin-8 (IL-8) (2/12). Across all of the studies, both males and females were included, and the mean age of participants ranged from 68 to 76 years. Eleven of the twelve studies performed exercise solely for the lower limbs, with one study using a whole-body exercise protocol [64].

**Table 7:** Cytokines

Study	Subjects	Age (years)	Exercise	Time points	Marker	Results			Other findings
						Time to peak	Magnitude of change	Time to recovery	
Przybyla et al. [48]	34 healthy males (17 young, 17 older)	32 ± 7, 71 ± 5	3 × 8 reps of bilateral leg press, leg curl, and leg extension 80% 1-RM. Plus 4th set to failure	Baseline, 72-h	IL-6 IL-1β IL-1RA IL-10 AMAC-1	N/A N/A N/A N/A N/A	↔ ↔ ↔ ↔ ↔	N/A N/A N/A N/A N/A	IL-1β, IL-10, IL-1RA significantly higher in older at rest, and IL-1β, IL-10, AMAC-1 increased two-fold in young but not older
Thalacker-Mercer et al. [66]	39 healthy adults (20 younger, 19 older)	37 ± 1, 73 ± 1	9 × 10 reps bilateral, concentric-eccentric knee extension 40% MIVC	Baseline, 24-h	IL-6 IL-8 TNF-α	N/A N/A N/A	↑ 0.34 pg/ml (24%)* ↔ ↔	N/A N/A N/A	IL-6 was also the only cytokine to change in young
Mathers et al. [69]	35 healthy adults (20 males, 15 females)	68 ± 1, 67 ± 2	3 × 12 reps of maximal isokinetic eccentric and concentric unilateral leg extension	Baseline, 2-h	IL-6 mRNA (men) IL-6 mRNA (men)	N/A N/A	↑ 0.193 AU (6433%)* ↑ 0.133 AU (1662%)*	N/A	Increased similarly in males and females
Funghetto et al. [61]	90 obese females	69 ± 6	7 × 10 reps eccentric bilateral knee extension isoinertial machine with a load corresponding to 110% of 10RM	Baseline, 0-, 3-, 24-, 48-h	IL-6	N/A	↔	N/A	
Patterson et al. [68]	7 healthy males	71 ± 7	5 sets of unilateral knee extensions 20% 1-RM to fatigue with or without blood flow restriction in a counterbalanced order	Baseline, 30-, 60-, 120-min	IL-6	N/A	↔	N/A	Blood flow restriction did not alter plasma IL-6
Buford et al. [57]	30 healthy adults (15 young, 15 older). Each group 5 females, 5 males	23 ± 4, 76 ± 5	150 reps unilateral eccentric contractions of plantar flexors 110% 1-RM	Baseline, 48-, 168-h	TNF-α	N/A	↔	N/A	
Tajra et al. [62]	90 obese females	69 ± 6	7 × 10 reps eccentric bilateral knee extension isoinertial machine with a load corresponding to 110% of 10RM	Baseline, 0-, 3-, 24-, 48-h	IL-6	0-h	↔(non-responders) ↑ 7.80 pg/ml (210%)*# (high responders)	48-h	‘Normal’ responders had no significant increase
Cornish et al. [64]	11 healthy males	72 ± 5	144 reps at 60% 1-RM OR 120 reps at 72% 1-RM OR 108 reps at 80% 1-RM of chest press, shoulder press, seated row, leg press, leg extension, and plantar flexion	Baseline, 0-, 3-, 6-, 24- 48-h	IL-6	6-h	↑ 0.49 pg/ml (28%)*#	24-h	RT intensity had no effect on IL-6 levels
Sorensen et al. [59]	19 healthy adults (11 young, 8 older). 4 females in young group	22 ± 2, 71 ± 7	300 reps maximal eccentric contractions of the knee extensors	Baseline, 24-, 72-h	IL-6 IL-1β MCP-1 MIG IP-10 I-TAC IL-7 IL-8 IL-13 GCSF	N/A N/A 24-h 24-h 24-h 24-h N/A 24-h N/A N/A	↔ ↔ ↑198 pg/ml (1191%)* ↑175 pg/ml (127%)* ↑195 pg/ml (1598%)* ↑63 pg/ml (322%)* ↔ ↑29 pg/ml (1140%)* ↔ ↔	N/A N/A Not recovered 72-h 72-h 72-h N/A Not recovered N/A N/A	
Pereira et al. [65]	28 healthy males	GG genotype: 71 ± 4 CC/CG genotype: 72 ± 4	10 × 7 reps of eccentric contractions of knee flexors and extensors	Baseline, 0-, 3-, 24-, 48-h	IL-6	N/A	↔	N/A	
Jensen et al. [70]	25 healthy males, 24 healthy females (12 young, 12 older)	70 ± 7 23 ± 3, 74 ± 3	Men: 5 × 12 reps (70% 1-RM) followed by 4 × 6 eccentric reps (110% 1 RM) Women: 2x [4 × 12 reps (70% 1-RM) followed by 4 × 4 eccentric reps (110% 1-RM) of unilateral knee extension]	Men: Baseline, 4.5-, 24-, 96-, and 168-h Women: Baseline, 120-h	TNF-α IL-10 IL-1β IL-1R COL1A1 Ki67 TNF-α IL-10 IL-1β IL-1R COL1A1 Ki67	N/A N/A 4.5-h 4.5-h 162-h 162-h N/A N/A N/A N/A N/A N/A	↔ ↔ ↑3.0-fold* ↑4.4-fold* ↑2.4-fold* ↑3.2-fold* ↔ ↔ ↔ ↔ ↔ ↔	N/A N/A 24-h 24-h Not recovered Not recovered N/A N/A N/A N/A N/A N/A	*Values only given relative to baseline (gene expression) Older had higher COL1A1 mRNA expression at 120-h after exercise compared to the young
De Sousa Neto et al. [63]	88 obese females	69 ± 6	7 × 10 reps eccentric bilateral knee extension isoinertial machine with a load corresponding to 110% of 10-RM	Baseline, 0-, 3-, 24-, 48-h	IL-6	N/A	↔	N/A	

*1-RM* one-repetition maximum, *RT* resistance training, *IL* interleukin, *min* minutes, *h* hours, *RT* resistance training, ↑ increase, ↔ no change

^#^Data extracted from figures

*Significantly different from baseline

In six of the ten studies that included IL-6 [48, 59, 61, 63, 65, 68], no significant increases in the cytokine were observed following resistance exercise. In two of the remaining four studies, increases in IL-6 ranged from 26 to 28%, at 24- [66], and 6-h [64] respectively, whereas in another study, IL-6 mRNA increased 1662–6433% 2-h post-exercise [69]. Lastly, in one study on 90 obese females, participants were grouped as ‘responders’ and ‘non-responders’ [62]. ‘Non-responders’ within this study had no significant increase in IL-6 after exercise, whereas the ‘responders’ group had significant increases of 210% from baseline. In all of the studies that IL-6 was found to increase, baseline levels were restored before 48-h.

No increases in TNF-α or IL-10 were observed after resistance exercise in older adults in any study. Jensen et al. [70] reported a threefold increase in IL-1β in men at 4.5-h, which returned to baseline at 24-h post resistance exercise. No increases were observed in women within the same paper, although blood samples were only taken at 120-h post-exercise in women, and they performed a different exercise protocol. No increases in IL-1β were observed in the other two of the three studies in which it was reported [48, 59], when it was measured at 24- and 72-h post-exercise. IL-8 increased by 29 pg/ml (1140%) at 24-h, and was not recovered at 72-h in a study by Sorensen et al. [59], but did not significantly increase in the study conducted by Thalacker-Mercer [66]. Other cytokines that were found to increase include MCP-1 [59], MIG [59], IP-10 [59], I-TAC [59], IL-1R [70], COL1A1 [70], and Ki67 [70].

## Discussion

### Age Differences in Recovery from Resistance Exercise

#### The Effect of Age on Exercise-Induced Muscle Damage in Men

Despite the small number of studies, the data suggest that older men may experience less EIMD than their younger counterparts, with four out of the five studies reporting at least one variable that had a significantly smaller magnitude of change post-exercise in the older group [45–47, 49]. More specifically, muscle strength was significantly less reduced in the older group in every study that reported it [45–47, 49]. No reported variable was significantly more changed in older adults’ post-resistance exercise compared to younger adults and it is currently unclear why this may be the case. This could be explained by lower absolute force produced by older adults during exercise in some of the studies, but this has not yet been investigated. The literature focussing on the time it takes older men to recover from resistance exercise is much less clear due to a full recovery of symptoms rarely being observed within the studies. For example, the study involving the longest follow up still did not see a full recovery of muscle strength at 240-h post-exercise [45]. This lack of observed recovery is consistent across every study and almost every variable, with the exception of muscle soreness in two of the studies [45, 46], and range of motion in one study [45]. This has implications for the prescription of training frequency and for exercise adherence, and should be taken into consideration when designing exercise interventions for older adults. Within these two studies, older men recovered muscle soreness and range of motion quicker than younger men, but this does not form strong enough evidence to suggest a differing recovery rate with age. This review was not designed to make any conclusions regarding the effect of age on EIMD, and a systematic review or meta-analyses will be needed to address this following the publication of more controlled trials. Indeed, the literature would benefit from further tightly controlled studies which aim to understand the length of time it takes older men to recover from resistance exercise, rather than only the effect of age on the magnitude of muscle damage.

It should also be acknowledged that three of the five studies used the same eccentric exercise protocol of the elbow flexors, and were published within a 2 year-period [45–47]. Whilst this is not an issue within itself, as it provides consistency within protocols, it should be considered when assessing the depth of the literature in this field. This is especially important as there is only one other study comparing physical function measures between young and older adults after resistance exercise, with the last study in the field reporting only the inflammatory response [48] to resistance exercise in men with age. It is therefore difficult to deduce the effect that age has on time to fully recover from damaging resistance exercise.

#### The Effect of Age on Exercise-Induced Muscle Damage in Women

Due to the limited number of studies of older women and exercise recovery, each marker of muscle damage has only ever been reported once in the literature. It is therefore difficult to draw conclusions on the effect of age on recovery in this population because of the lack of available data. However, initial data suggest that whilst age appears to convey some protection against EIMD in older men, the opposite may be true for older women. Previous work has suggested a protective effect of oestrogen on EIMD [71, 72], which could provide an explanation for this trend. Indeed, oestrogen is typically much lower in older post-menopausal women than young women, and this reduction in hormone levels could explain the impaired exercise recovery rates in older compared to younger women. Although this has not yet been investigated, a previous systematic review [72] found five studies that reported markers of EIMD in young women who were or were not taking oral contraceptives [73–77]. In the one study in which endogenous oestrogen was higher prior to exercise in the oral contraceptive group, a lower CK response was reported post-exercise compared to the menstrual cycle group [77], suggesting a potential protective effect of oestrogen against EIMD. However, like the present review, no conclusions could be drawn due to a relatively small number of varied studies. Hence, it may be that different hormonal changes with age between sexes may be a greater determinant of how older adults recover from resistance exercise, rather than age itself. It may therefore be beneficial to the literature in both men and women to investigate a spectrum of age groups to track any changes in exercise recovery across the lifespan. An important aspect of this research would be the assessment of the effects of major physiological milestones (e.g. the menopause) on exercise recovery alongside the progression of chronological age.

### Symptoms of Exercise-Induced Muscle Damage

#### Physical Function

There is no clear consensus on the magnitude of the effect, or on the time it may take older adults to recover from muscle damaging exercise. Both outcomes are relevant when designing training programs for previously untrained older adults. The lack of consensus within the literature on these variables likely stems from the variation in study protocols that have been highlighted by this review, mainly differences in the exercise protocols, the muscle groups investigated, and the time-points when measures have been collected. For example, time to complete the TUG test varied between the two studies that reported it, with one reporting a 2% increase in time [12], and the other 18% [54]. This is perhaps unsurprising given that different muscle groups were used across the two studies. Similarly, studies that assessed function of the elbow flexors tended to report larger decreases in strength than those that assessed function of the lower limbs. Decreases in elbow flexor MVC after exercise ranged from 42 to 49%, whilst decreases in plantar flexor and knee extensor strength ranged from 9 to 36%. This is not novel information, as the differences in the susceptibility of muscle groups to EIMD have previously been reported with the upper limbs generally incurring more damage than the lower limbs, possibly due to muscle fibre characteristics and daily exposure to eccentrically biased actions [13, 78, 79]. However, this lack of consensus within the literature is not exclusively a result of differing muscle characteristics, but may also have been affected by exercise intensity, volume, individual characteristics of the participants, and study characteristics. Greater alignment of study protocols in the future, or a more considered approach of choosing an exercise protocol would be beneficial to determine the real-world effects of resistance exercise on older adults and allow more informed recommendations to be made. Indeed, it is unlikely that practitioners would prescribe a resistance exercise programme consisting of maximal eccentric contractions that are intended to cause maximal muscle damage. The outcome measures used to represent function are generally consistent, with 13/15 studies measuring MIVC/MVC, a measure of muscular strength. However, when working with older adults, it may be wise to consider including additional measures of physical function alongside muscle strength which may be more clinically meaningful, such as falls risk or the TUG test, to ensure findings are also applicable to the population.

Similarly, of the studies reporting physical function, few were of long enough duration to observe a convalescence of muscle strength and make recommendations for training frequency. It is unclear if this is due to a lack of ecological validity within the studies (i.e. the training prescribed induced much greater damage than would usually be observed in training), or if this is the true time it takes older adults to recover. The longest study was 264-h in duration, and recovery of concentric 1-RM was not observed until 168-h [52]. However, not all studies observed full recovery. In a study that was 240-h in duration, MIVC of the elbow flexor after eccentric exercise was still not recovered to baseline by the end of the study [45]. Similarly, the next longest study had participants return at 168-h, but maximal contraction of the plantar flexors was also not recovered at this time [57]. In three of the studies [12, 55, 59], peak decreases in function occurred immediately after exercise and function was recovered by 24-h. It is likely that where a peak decrease in muscle function was recorded immediately after exercise, neuromuscular and metabolic fatigue were greater contributors than muscle damage at this time point. Large variation, a lack of studies that observed full recovery, and a lack of uniformity with time points for outcome measures means it is therefore unclear at exactly what rate older adults recover physical function. Without this information, it is difficult to recommend training frequencies or volumes that will both limit negative consequences of EIMD and ensure optimal adaptation time in between exercise bouts. Additionally, if there is residual fatigue from previous exercise, it is likely that the quality of the training session could be affected, or adherence may be reduced. Hence, if older adults do take approximately a week to recover, practitioners may consider prescribing exercise in 2- or 3-week blocks allowing more time between sessions, rather than using the traditional weekly cycles. Practitioners may also wish to consider adapting the volume or intensity of resistance exercise sessions, or adopting a body part-split approach to ensure physical activity guidelines of strength training two times per week are being met. Future studies seeking to characterise exercise recovery in older adults should extend the time that data are collected past 168-h at the least, but may wish to consider extending beyond 240-h. More importantly, focus should be given to characterising exercise recovery in response to the various training variables that may be manipulated to ensure optimal exercise prescription.

#### Muscle Soreness

There is a considerable body of literature assessing the effect of resistance exercise on muscle soreness in older adults. The magnitude of change and time to peak change are relatively consistent across the studies, with all studies reporting peak changes at 24–48-h. However, due to discrepancies in the visual analogue scales used, it is difficult to provide an absolute value for the magnitude of change. Nevertheless, most studies assessing muscle soreness in older adults following resistance exercise reported only mild increases in soreness ratings, although it is unclear why this may be, and more work is needed to understand this.

There is some evidence that pain perception could decrease with ageing [80] and thus, it is possible that the levels of muscle soreness within these studies have been under-reported. Indeed, three of the studies that compared muscle soreness with younger adults found that self-reported muscle soreness was significantly lower in the older group [45, 46, 57]. In addition to a possible systemic under-reporting of soreness compared to younger adults, there is also large individual variation in the interpretation of the visual analogue scale [81, 82]. Caution should therefore be taken when analysing subjective muscle soreness, as it may only be useful when comparing intra-individual variation within a study, rather than drawing conclusions from absolute group values. Most of the studies reporting muscle soreness in older adults have used an eccentric exercise protocol, with only two using a concentric protocol. Whilst eccentric protocols are common practice to induce muscle damage for research purposes, they may not directly translate to the exercise sessions performed in a real-world setting, as they tend to cause greater soreness and damage [28].

It has been suggested previously that older adults may be deterred from completing a resistance exercise programme if expectations that muscle soreness will be experienced are not set from the beginning [3]. The rationale for this is that they may confuse muscle soreness with injury or believe they will experience this soreness after every exercise bout. If the current data surrounding muscle soreness in older adults are accurate, and older adults have not under-reported soreness ratings, it is likely that pain experienced from usual resistance exercise will be mild, especially if performing mainly concentric-based exercise protocols. Hence, it is unlikely that muscle soreness would prevent older adults from engaging in a structured resistance exercise programme. Nevertheless, muscle soreness is highly individual, and educating older adults prior to beginning an exercise programme may aid adherence in some older adults.

#### Falls Risk

There is a distinct lack of literature seeking to understand the acute effect of resistance exercise on falls risk in older adults. Despite there only being two studies within this area [54, 60], both indicate a potential detrimental effect on postural stability, which has long been associated with an increase in fall incidence [83, 84]. Similarly, several contributors to falls risk (e.g. muscle strength and power) are also compromised in the presence of EIMD and may contribute to falls risk. The earliest study provided evidence of decreased postural control after a single bout of resistance exercise using stabilogram-diffusion analysis [60], and the latest study, some 16 years later, showed an increase in COP sway area of 36% after damaging exercise of the calf muscles [54]. Interestingly within the most recent study, fear of falling was also shown to increase after a bout of resistance exercise. Together the studies begin to provide important data regarding postural stability after resistance exercise in older adults. However, significant further research is needed within this area to fully understand the intricacies of how muscle damage and fatigue may affect falls risk, and whether reduced postural stability is a normal response to resistance exercise across all age-groups.

Postural stability and the risk of falls is a significant topic in geriatrics but so far there are very limited data on the effects of exercise on these parameters. The effects of resistance exercise on acute falls risk appears to be somewhat of a ‘blind spot’ within the literature. It is possible that this is due the topic sitting within two historically separate fields of literature. Indeed, clinical research often does not go as far to produce research on the effects of exercise, whilst exercise science often does not consider the clinical implications of an exercise prescription for older adults. In an age where resistance exercise is being increasingly more commonly prescribed for older adults, this presents a pressing need for clinicians and exercise scientists to collaborate and ensure the best care for older generations.

### Biological Markers of Muscle Damage

#### Circulating Muscle Proteins

The circulating muscle proteins that have been reported following resistance exercise in older adults are CK, myoglobin and lactate dehydrogenase. Potentially due to the relatively low cost of assays required for quantification, the most commonly reported of these is CK, with 14 of the 15 studies including the measure in their outcomes. However, there is no clear consensus across this literature on the magnitude of change or the temporal characteristics of CK post-exercise. This is similar to findings from research in younger adults post-exercise [32] where CK is also widely variable in both magnitude of change and the time course of fluctuations. When comparing the younger and older age groups, there is some evidence that CK increases to a greater extent in younger adults than older adults [45, 46], but other studies show no difference between the groups [49, 50].

The extent to which CK increases after exercise is dependent on individual factors, as well as environmental, and exercise variables [32, 85]. The large variation of CK in studies across all age groups should be seen as a major limitation in exercise recovery research. Indeed, researchers should question whether assessing CK after exercise is an efficient use of resources, as often variation is too great to confer statistical significance between groups. Given the practical and ethical implications of collecting blood to measure CK, it should also be considered whether it is a useful measure. Whilst CK levels do generally increase after EIMD, this is not always parallel to the magnitude of muscle fibre damage [86], and does not directly correlate to muscle function or other symptoms of EIMD that may be of more relevance to the individual [33]. Hence, CK may be better used as a binary marker to determine the presence of EIMD or membrane disruption. It may be of greater use to the literature to focus on functional outcomes, or outcomes that may more directly inform the state of recovery when conducting research in older adults. The conclusions made for CK within this review also extend to myoglobin, but not to lactate dehydrogenase, for which there are not enough data to determine if this measure is valid or reliable.

#### Cytokines

Similarly to circulating muscle proteins, there is no clear consensus on the effect of resistance exercise on the acute cytokine response in older adults despite numerous studies reporting this outcome. Interleukin-6 (IL-6), the major cytokine associated with the acute post-exercise inflammatory response [87], was the most commonly reported, and was generally a secondary outcome, but did not significantly increase in over half of the studies. Other cytokines that have been reported also do not reliably increase after resistance exercise in older adults. This finding appears to be similar for both inflammatory and anti-inflammatory cytokines. Due to the complexity of the overall post-exercise cytokine response, the non-specificity of cytokines to skeletal muscle, and the large inter-individual variability for each cytokine [88], it is unlikely that this is useful to measure when aiming to compare the EIMD response amongst older adults. As cytokines are important mediators of chronic exercise-induced adaptations, it would be more pertinent to consider the effect of resistance exercise on these markers in older adults using specific, large scale studies. Studies that are designed to characterise symptoms of EIMD, and where the sample size is likely not large enough to convey the required statistical power for assessing the cytokine response, should refrain from including this outcome on both ethical and practical grounds.

### Scoping Review Limitations

The format of a scoping review presents some limitations. To achieve our objectives of mapping the state of the current literature surrounding exercise recovery in older adults, broad search terms were used which resulted in a large number of studies being identified with varying outcomes. This has created a large data set, and hence, our review can only provide a brief overview of each facet of recovery from resistance exercise in older adults.

## Conclusion

Chronic adaptations to resistance exercise are well documented, but the acute effects of resistance exercise, specifically the magnitude and time-course of EIMD in older adults, are less clear. The process of EIMD after resistance exercise has been extensively reviewed amongst younger adults, but original articles exploring this phenomenon in older adults are sparse. From the studies that have been conducted, it is hard to draw definitive conclusions about the effect of resistance exercise on physical functioning in the following days due to variations in study protocols and outcome reporting. Similarly, research into biological markers of muscle damage in older adults is limited and inconsistent, and their validity for assessing the magnitude of EIMD should be considered. Research surrounding the presence of delayed onset muscle soreness in older adults is more consistent but still fails to answer why older individuals generally report less soreness than their younger counterparts. Across all measures of EIMD, data in females are lacking when compared to males, and rectifying this discrepancy should be a focus of future studies considering the large proportion of the population that this represents. A greater understanding of how resistance exercise affects function in the following days is essential to inform better exercise prescription and the formation of suitable exercise recovery strategies for older adults.

## Data Availability

All data collated during this study are included in the published article.

## Abbreviations

1-RM: One-repetition maximum
CK: Creatine kinase
COP: Centre of pressure
EIMD: Exercise induced muscle damage
IL: Interleukin
LDH: Lactate dehydrogenase
Mb: Myoglobin
MVC: Maximal voluntary contraction
TNF: Tumor necrosis factor
TUG: Timed up-and-go
